# Aboveground and belowground trait coordination across twelve boreal forest tree species

**DOI:** 10.1038/s41598-024-84162-0

**Published:** 2025-01-03

**Authors:** Clydecia M. Spitzer, Sandra Jämtgård, Marcus J. Larsson, Michael J. Gundale

**Affiliations:** 1 Department of Forest Ecology and Management, Swedish University of Agricultural Sciences, Skogsmarksgränd, Umeå, 901 83 Sweden; 2 Department of Forest Genetics and Plant Physiology, Swedish University of Agricultural Sciences, Linnéus väg 6, Umeå, 901 83 Sweden

**Keywords:** Boreal forest, Trait coordination, Trait-environment relationships, Root traits, Leaf traits, Within-site variation, Ecology, Plant sciences, Ecology

## Abstract

The existence of trait coordination in roots and leaves has recently been debated, with studies reaching opposing conclusions. Here, we assessed trait coordination across twelve boreal tree species. We show that there is only partial evidence for above-belowground coordination for “fast-slow” economic traits across boreal tree species, i.e., while N content in leaves and roots were positively correlated, as well as dry matter content, root dry matter content and leaf N had no significant relationship. For resource acquisition traits (i.e. related to light capture and nutrient uptake) we did not find strong evidence for trait coordination, as specific root length and specific leaf area were not positively correlated. We further show that site only explained between 0 and 7% of the total trait variation, while within-site variation contributed substantially to the total trait variation for a large number of traits (1.6–96%), and more so for morphological root traits than leaf traits. This likely influences the strength of above-belowground trait coordination found across species in our study. Understanding sources of trait variation and above-belowground trait relationships can contribute to improving global and regional C cycling models. However, fine-scale environmental variability should be accounted for given its importance for driving trait variation.

**Supplementary Information:**

The online version contains supplementary material available at 10.1038/s41598-024-84162-0.

## Introduction

Plant functional traits and their values are important for understanding soil carbon (C) and nitrogen (N) cycling at various scales^1,2^. One major hindrance for their inclusion into global C cycling models has been our limited understanding of trait co-variation and coordination across plant structures. An understanding of trait relationships is important for reducing redundant traits in models, and for the substitution of one trait with another in the absence of data on a particular trait. Our understanding of trait relationships in various plant organs has markedly improved over recent decades. Earlier trait-based research was heavily focused on leaf traits^3–5^ and ultimately led to a consistently observed gradient of aboveground traits, referred to as the “leaf economics spectrum”^5,6^. This gradient provided evidence for a trade-off between fast growth (with higher N content) and leaf longevity. Later research by Diaz et al.^7^ expanded the leaf economics spectrum into two gradients, one being the leaf economics spectrum, and the other incorporating the size of the plants and seed mass.

Regarding belowground traits, it was first hypothesized that root traits would have a similar one-dimensional spectrum, parallel to the leaf economics spectrum^8,9^. However, Bergmann et al.^10^. later found strong evidence for a “root economics space” in their global meta-analysis, and this general pattern of interspecific trait variation has since been observed in multiple studies^11,12^. The “root economics space” consists of two axes. The first axis (i.e. the collaboration gradient) is related to the collaboration of plants with mycorrhizal fungi for nutrient acquisition, i.e. with one end of the gradient consisting of roots with higher specific root length and the other end of the gradient consisting of plants with thicker fine roots. The second axis of the root economics space (i.e. referred to as the “conservation gradient”), is related to trade-offs between fast growth and metabolism (with higher nutrient content) versus higher root tissue density. These recent insights into root trait variation have improved our understanding of interspecific root trait variation, and have further opened up new avenues to explore the extent to which aboveground and belowground traits are coordinated^13–15^.

Some evidence for the correlations of aboveground and belowground traits has existed for over two decades^4,9,13,16^. In addition, Reich^15^ hypothesised that selection along trait trade-off axes drive species to have convergence to fast, medium or slow strategy of resource acquisition and processing across all organs. Indeed, a fast-slow spectrum of economic strategies has been observed in both the “leaf economics spectrum” and the “root economics space.” However, there is currently an ongoing and unresolved debate based on meta-analyses as to whether above and belowground traits are coordinated along a fast-slow spectrum^11,17–19^. The debate has been dominated by disagreements regarding the presence of coordination of N in leaf and root tissue, which is at the fast end of the spectrum. Tissue density, which is at the slow end of the spectrum, was included in the meta-analysis of Weigelt et al.^17^. The study found a strong correlation between analogous leaf and root traits (i.e. tissue N content and tissue density) at each end of the “fast-slow spectrum”. However, it remains unclear whether traits at each end of the fast-slow spectra are inversely related. Further, it remains unclear whether above- and belowground traits are coordinated along a resource acquisition gradient, such as the relationship between the collaboration gradient in the root economics space and specific leaf area. Given the disagreement over the presence or strength of these potential above- and belowground trait relationships, new research approaches and data are needed to evaluate above-belowground trait relationships.

Further, several factors might make it difficult to identify coordination between above- and belowground traits across species, such as trait variation that occurs within and between sites, due to genetic and environmental variability. In addition, global trait meta-analyses on trait coordination typically do not consider intraspecific variation. Therefore, study designs allowing for quantification of the contributions of site differences (based on environmental differences), interspecific and intraspecific variation to total variation of analogous traits may serve as a valuable tool to investigate the influences on above-belowground trait coordination. Indeed, one key remaining gap of knowledge highlighted by Weigelt et al.^17^ is a need for understanding the contributions of interspecific and intraspecific variation in plant tissues, and whether these ranges differ for above- and belowground traits. In terms of site effects on traits, leaf mass area has been found to be modestly positively or negatively correlated with a number of macroclimatic variables in a global meta-analysis^5^. Belowground, some root traits such as specific root length and specific root area have been found to vary with soil nutrient availability and several macroclimatic variables, while others have been unresponsive or inconsistent across studies^20–24^. Recently, it has been observed that site has little influence on aboveground trait variation across species^25^, but it is not understood whether site influences analogous root and leaf traits differently.

Moreover, the contribution of intraspecific trait variation to total trait variation of analogous root and leaf traits is not understood. Studies have shown that within-species trait variation can contribute anywhere from a little to a large percentage of total trait variation in leaves and roots^26–28^. Studies have not partitioned the contribution of intraspecific trait variation to total trait variation for analogous root traits relative to leaf traits. These relationships may influence the strength of above-belowground trait coordination, as disproportionate contributions of intraspecific variation to either above- or belowground traits may obscure above-belowground trait coordination patterns. In terms of species identity, studies have found that it could contribute the most to total trait variation^28–30^, likely due to the importance of phylogenetic relatedness in driving root trait variation^16^. The use of multiple common garden experiments provide the possibility to disentangle the relative importance of between- and within-site variability, as well as species identity in explaining trait variation^31,32^.

**Fig. 1:**
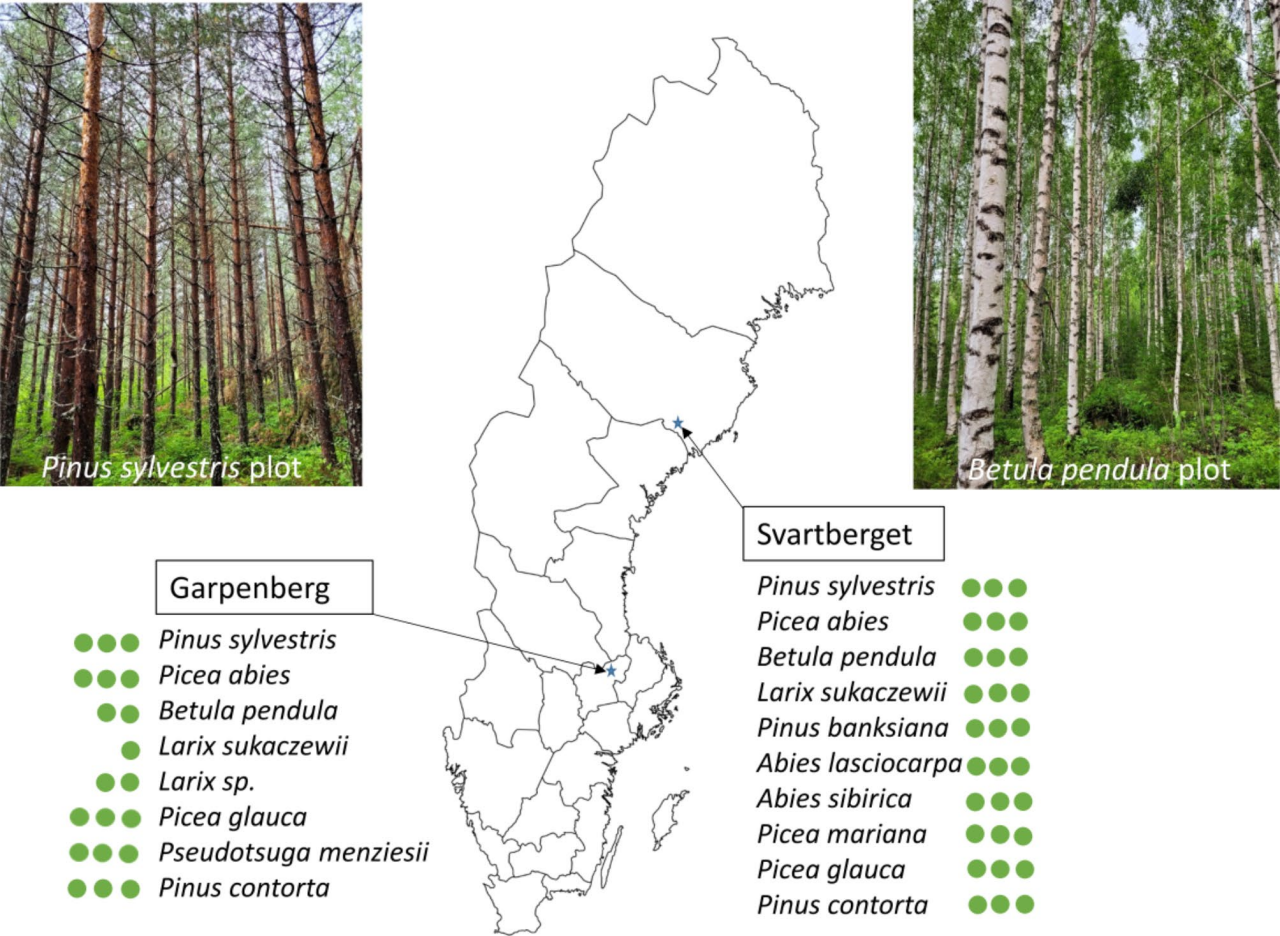
Map showing the locations of the two field sites in Sweden. Three replicate blocks are present at each site, with each block consisting of plots (0.1 ha) with monocultures of each tree species planted at the site. Tree species present at each site are listed and the number of green dots indicate the number of replicate plots of each species that are found at the site (30 plots at Svartberget and 20 in Garpenberg). Photos are one replicate plot of two species (photo source: C. Spitzer). Map source: www.vemaps.com.

In this study we used two common garden experiments, one in northern and one in central Sweden, and each with replicated blocks of monocultures of tree species, to investigate whether above- and belowground traits were coordinated across 12 boreal tree species, and to quantify the influence of between- and within site variation on these relationships. The study included the most dominant tree genera in boreal forests, which are significant from a global perspective because boreal forests cover approximately 17% of the terrestrial land surface area, and thus these genera play a very important role in the global C cycle by promoting a strong biome C sink^33–35^. We tested the following hypotheses: (i) Fast-slow strategies will be aligned above- and belowground to form a trait trade-off axis, i.e. root and leaf N content will be positively correlated with each other, and N content in roots will be negatively correlated to dry matter content in leaves and vice versa. We expect this because both the “root economics space” and the “leaf economics spectrum” consist of a fast-slow conservation gradient, and selection along trait trade-off axes could drive species to have a convergence to fast, medium or slow strategy of resource acquisition and processing across all organs^15^; (ii) Root resource acquisition traits related to “the collaboration gradient” will form a second axis consisting of specific root length and specific root area at the one end^12^, and root diameter at the other^10,17^. Further, we anticipate that specific leaf area and specific root length, which are analogous traits related to resource acquisition (i.e., light capture in leaves and nutrient uptake in roots), will either have positive or weak relationships along the root collaboration gradient^4,9,13,17,36^; (iii) Species identity will be the dominant contributor to total trait variation, followed by site and intraspecific trait variation. We further expect that the proportional contribution of these factors to trait variation will be similar for above- and belowground traits that are strongly coordinated. Testing these hypotheses in combination will lead to an improved understanding of the degree to which above- and belowground traits are coordinated in boreal tree species, and identify key controls that influence the strength of these relationships (Fig. 1).

## Results

**Fig. 2:**
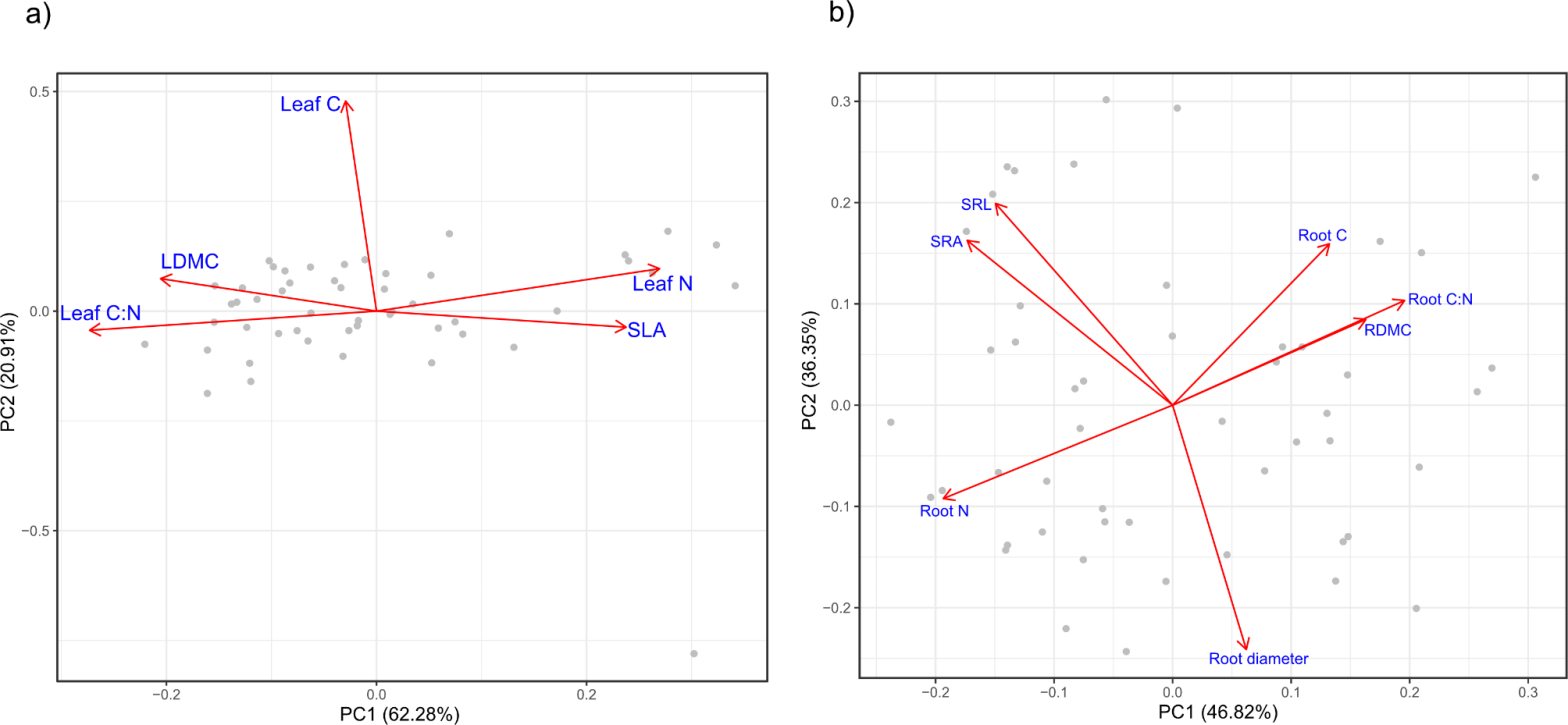
Principal Component Analysis of leaf and fine root traits across all species. **a** Leaf trait variation only. **b** Fine root trait variation only. Traits corresponding to the bi-plot arrows are shown in blue font: leaf carbon content (Leaf C); leaf nitrogen content (Leaf N); specific leaf area (SLA); leaf dry matter content (LDMC); leaf carbon to nitrogen ratio (Leaf C: N); average root diameter (Root diameter); root nitrogen content (Root N); specific root area (SRA); specific root length (SRL); root carbon content (Root C); root carbon to nitrogen ratio (Root C: N), and root dry matter content (RDMC).

Regarding aboveground trait variation, leaf N and specific leaf area were positively related, and both traits were negatively related with leaf C: N ratio and leaf dry matter content. These traits aligned along the first principal component axis and contributed to 62.3% of the total variation in aboveground traits (Fig. 2). The second principal component explained 20.2% of the total variation and was influenced by leaf C content.

For belowground traits, the first principal component explained 42.8% percent of the variation in traits, and was primarily comprised of root C, root C: N ratio, and root dry matter content at the one end, and root N at the other. The second axis explained 36.4% of the variation, with specific root area and specific root length being positively related, and each of these negatively related to root diameter.

### Root and leaf trait coordination

**Fig. 3:**
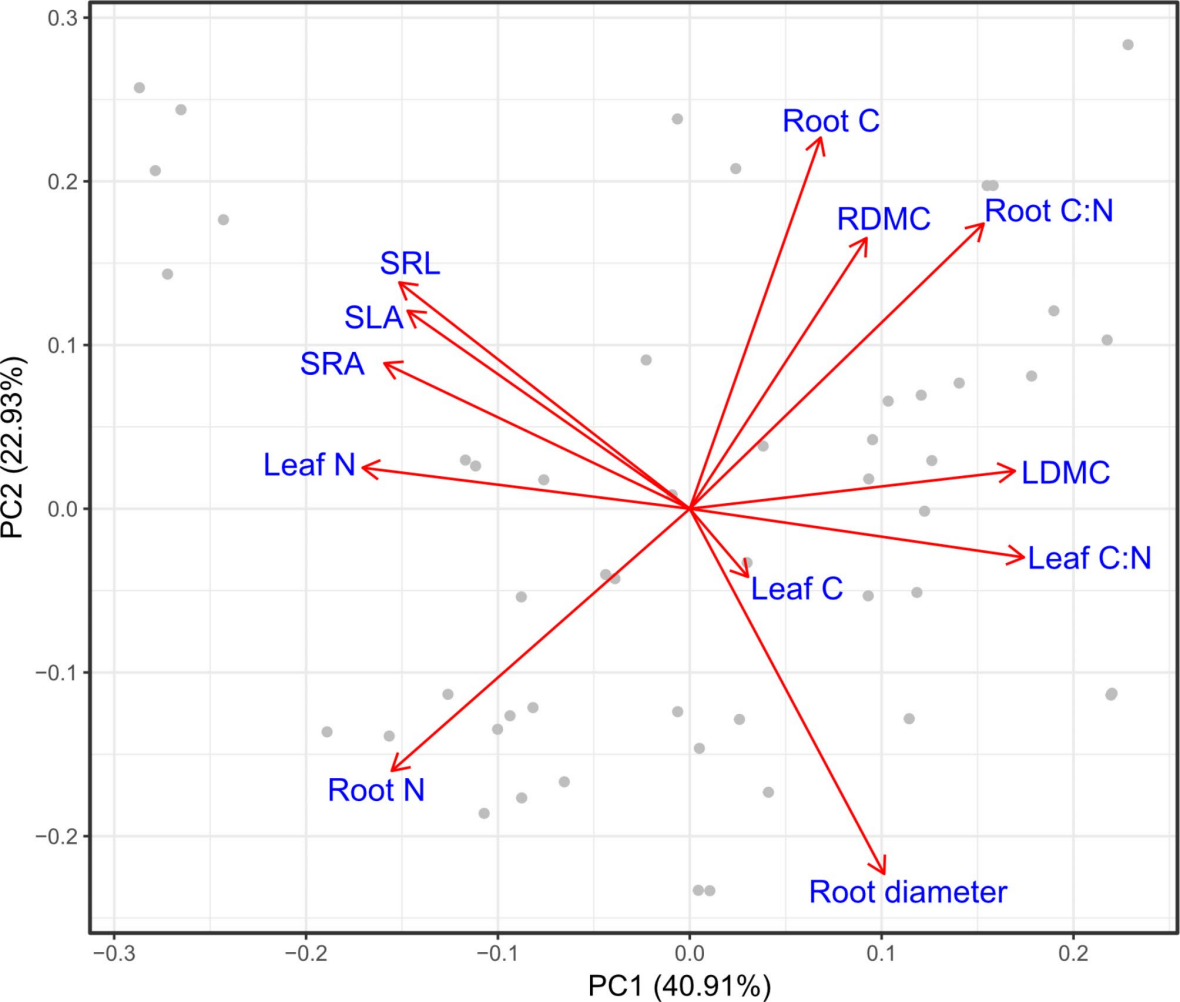
Principal Component Analysis of above- and belowground plant traits across all species. Traits corresponding to the bi-plot arrows are shown in blue font: leaf carbon content (Leaf C); leaf nitrogen content (Leaf N); specific leaf area (SLA); leaf dry matter content (LDMC); leaf carbon to nitrogen ratio (Leaf C: N); average root diameter (Root diameter); root nitrogen content (Root N); specific root area (SRA); specific root length (SRL); root carbon content (Root C); root carbon to nitrogen ratio (Root C: N), and root dry matter content (RDMC).

**Fig. 4:**
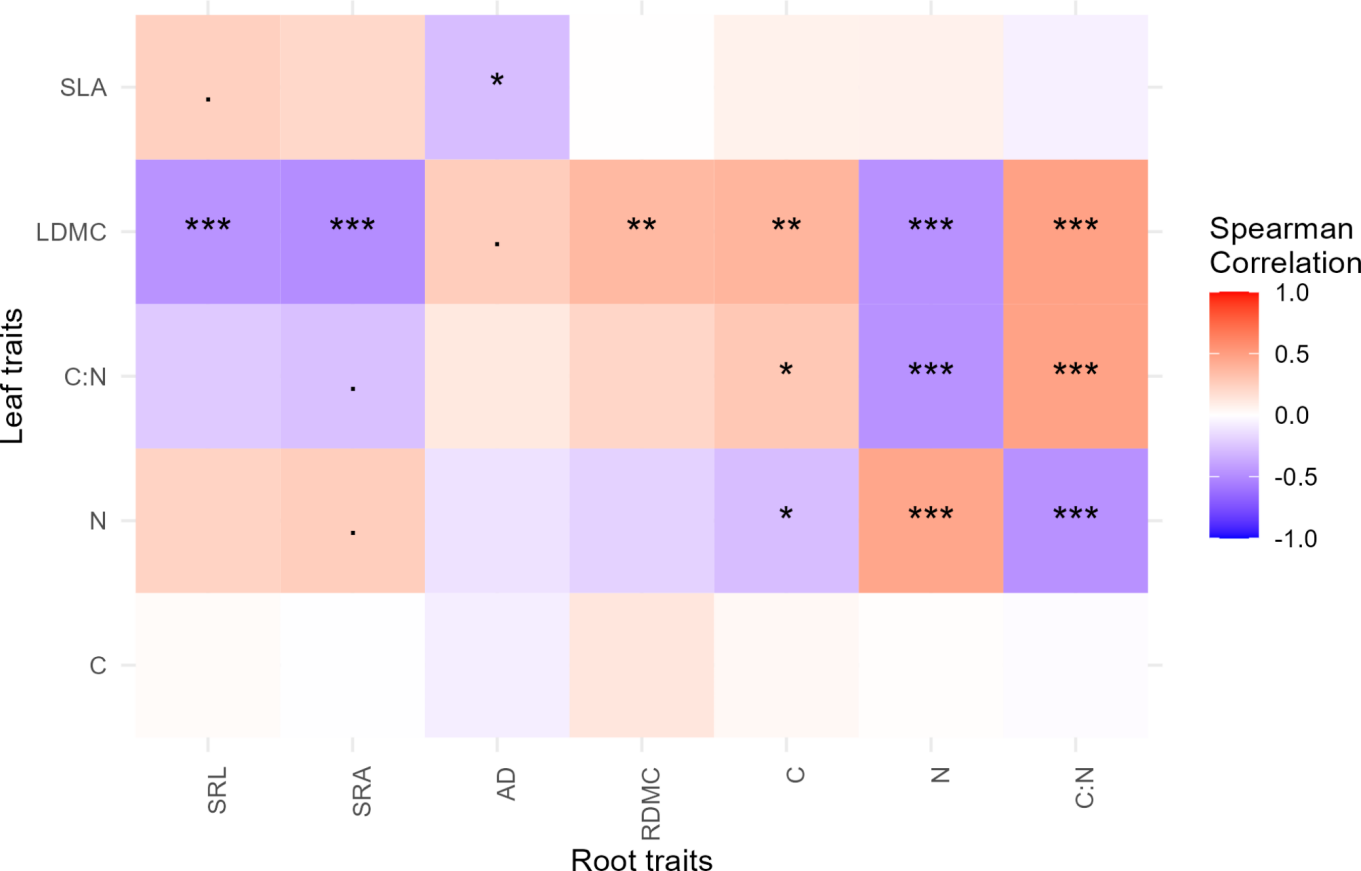
Spearman’s rank correlation matrices between fine root traits and leaf traits across all species. The six leaf traits are leaf carbon content (C); leaf nitrogen content (N); specific leaf area (SLA); leaf dry matter content (LDMC); leaf carbon to nitrogen ratio (C: N). The eight fine root traits are average fine root diameter (AD); root nitrogen content (N); specific root area (SRA); specific root length (SRL); root carbon content (C); root carbon to nitrogen ratio (C: N), and root dry matter content (RDMC). Asterisks indicate statistical significance (* indicates *P* ≤ 0.05; ** indicates *P* ≤ 0.01; *** indicates *P* ≤ 0.001).

With root and leaf traits from both common garden sites combined into a single PCA 63.8% of the total variation was explained in the first two PCA axes. The first axis explained 40.91% of the total variation, whereas the second axis explain 22.9% of the variation. We found that leaf and root N were positively correlated with each other, and negatively correlated with dry matter content in both roots and leaves (Fig. 3; *r* = 0.46; Fig. 4). We found a positive relationship between dry matter content in leaf and root tissue (Fig. 3; *r* = 0.37; Fig. 4), with the relationship being stronger at the Garpenberg site (Supplementary Fig. 1). Tissue C: N ratio was positively related to C content and dry matter content in roots and leaves (Fig. 3).

Specific leaf area, specific root length and specific root area were positively associated with each other (Fig. 3). However, after conducting a Spearman’s rank correlation analysis with Holm’s posthoc corrections for multiple comparisons, specific leaf area had a marginally non-significant relationship with specific root length and no significant relationship with specific root area (Fig. 4). Specific leaf area had a significant negative relationship to average root diameter (*r* = -0.28; Fig. 4). Leaf N was positively related to specific root length, specific root area and specific leaf area (Fig. 3), but the relationship between leaf N and specific root length and specific root area was not significant after accounting for multiple comparisons (Fig. 4). Of these relationships with leaf N, the stronger positive correlation was with specific root area (*r* = 0.26; Fig. 4). Root dry matter content did not have a significant correlation with leaf N.

### Sources of trait variation

**Fig. 5:**
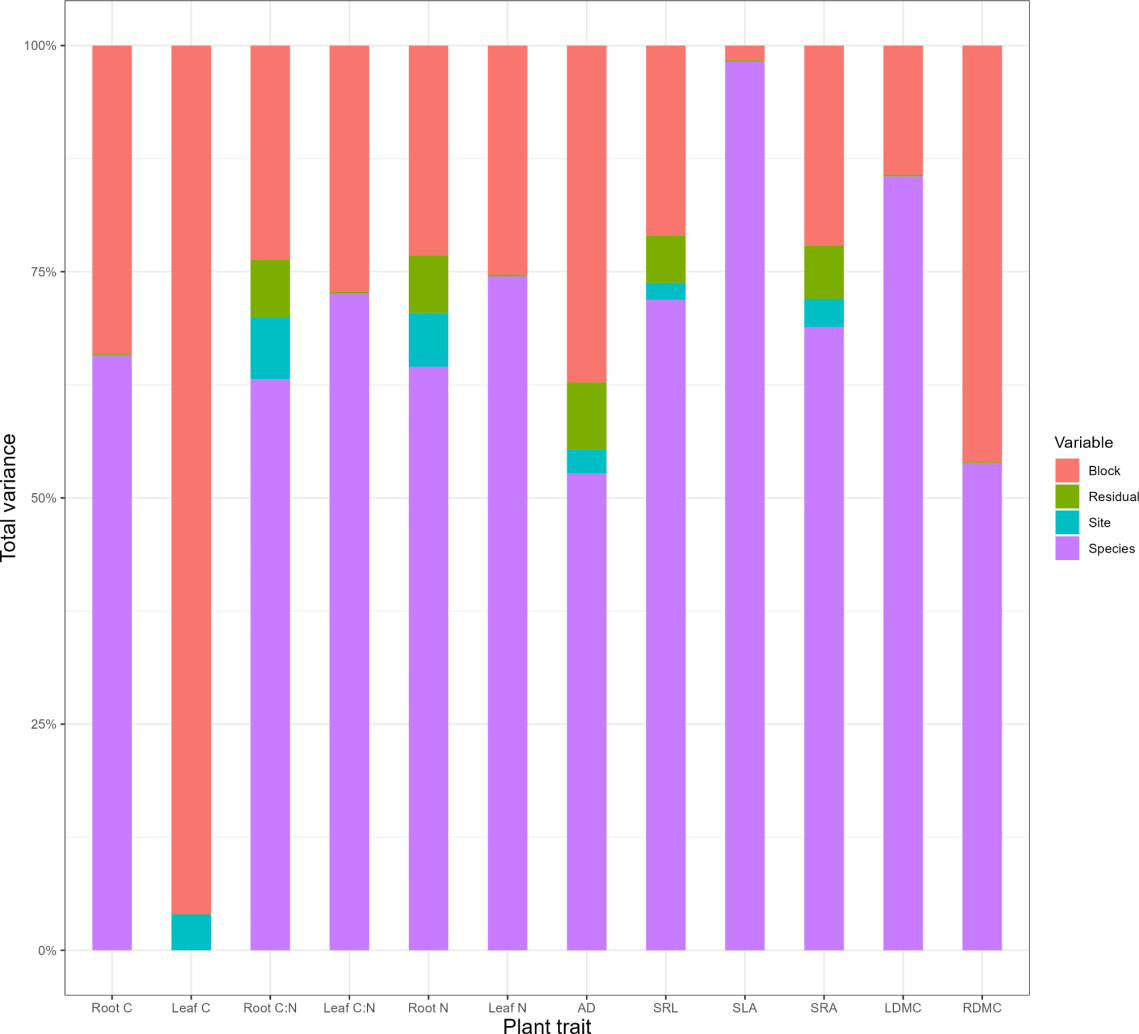
Variance decomposition for each trait across all tree species. Bars are the total trait variation for each plant trait. The sub-bars shows the percentage of the total variation for each trait explained by species (purple), site (blue), variation between blocks within a site (peach) and residual variation (green). The fourteen plant traits are root carbon content (Root C); leaf carbon content (Leaf C); root carbon to nitrogen ratio (Root C: N); leaf carbon to nitrogen ratio (Leaf C: N); root nitrogen content (Root N); leaf nitrogen content (Leaf N); average fine root diameter (AD); specific leaf area (SLA); specific root length (SRL); specific root area (SRA); leaf dry matter content (LDMC), and root dry matter content (RDMC).

With both sites combined, the total percentage variation in trait values explained by the first two principal component axes is 63.8% (Fig. 3); whereas, the percentage variation explained by the first two axes at the individual sites was higher, i.e., 67.6% in Svartberget and 68.6% at Garpenberg (Fig. S1). In terms of site effects on total trait variation, we found that site explained between very little to no variation depending on the trait considered (Fig. 5). No trait variation was explained by site for leaf C: N ratio, dry matter content, N content and specific leaf area. Leaf C was the only aboveground trait that had some variation explained by site (i.e. 4%). For root traits, site explained between 2% and 7% of the total variation, with the exception of root dry matter content and C content which had none of the total variation explained by site. Root N content and C: N ratio had 6% and 7% of the total variation explained by site, respectively.

Species generally explained the largest proportion of the total variation of traits, ranging from 53 to 98%, in all traits except leaf C, where it did not explain any of the total variation. Meanwhile, for leaf traits, the total trait variation explained by within-site variation ranged from 1.6% for specific leaf area to 96% for leaf C (Fig. 5). For root traits, the smallest proportion of the total trait variation explained by within-site variation was 20.9% for specific root length and 46.1% for root dry matter content. In terms of analogous traits root and leaf traits, the proportion of total variation explained by intraspecific variation, site and interspecific trait variation differed, even when those traits were positively correlated (Table S1; Figs. 4 and 5).

## Discussion

Our study disentangled the contribution of species identity and the variation between and within sites to total trait variation of twelve above- and belowground traits across a large number of boreal tree species. We show that site explains only a small proportion of the total variation of all measured leaf and root traits, whereas species identity explains the largest proportion of that variation. Further, we provide new evidence for a large contribution of within-site variation to total trait variation of both root and leaf traits across boreal tree species. Particularly, we show for the first time that the contribution of within-site variation to total variation was generally larger in analogous morphological root traits than leaf traits for boreal tree species. In addition, we found that a portion of analogous fast-slow conservation traits were coordinated above- and belowground, therefore providing partial evidence for a fast-slow trade-off strategy across tissues. However, our study did not find strong evidence for trait coordination related to resource acquisition, in that a positive correlation between specific root length and specific leaf area was not found, whereas average diameter was negatively correlated to specific leaf area.

In regards to our first hypothesis, we predicted that fast-slow strategies will be aligned in above- and belowground plant structures. We found partial support for this hypothesis, as dry matter content in leaves and roots were indeed strongly positively correlated with each other, and there was a strong positive relationship for N content in the two tissue types. The positive correlations between C: N ratios in roots and leaves is similar to those reported by Valverde-Barrantes et al.^16^. in temperate tree species. The results related to N are similar to those reported by Weigelt et al.^17,18^. These findings are also in line with the hypothesis by Reich^15^ that species evolve convergent fast-slow strategies across all tissues. However, in our study, leaf dry matter content was negatively correlated to root nitrogen content, but root dry matter content did not have a significant negative correlation with leaf N content. This finding contrasts with those of Weigelt et al.^17^. , where root tissue density, a trait similar to root dry matter content^37^, was found to be negatively correlated to leaf N content. Nevertheless, it points to a decoupling of root tissue conservation strategies and fast strategies in leaves in boreal forests, as short growing seasons promote increased photosynthesis to maximize C gain^38^ (with leaf N being positively correlated with photosynthetic rates^39^), whereas roots appear to be constructed to maximize tissue longevity^40^. In addition, our study classified fine roots as < 2 mm, which may include transport roots. While this root diameter class is frequently selected for root trait studies^30,41^, the potential inclusion of some transport roots might have contributed to different results in our study compared to those of Weigelt et al.^17^. Interestingly, leaf dry matter content has significant correlations with six of the seven measured root traits, and is therefore a potential trait for inclusion in models related to ecosystem functioning both above- and belowground. Indeed, several studies have found this to be a reliable trait in models predicting net primary productivity^42,43^. Trait coordination above- and belowground may allow for more reliable predictions of soil C cycling, as fast traits (e.g. higher tissue N content) generally have faster turnover rates both in roots and leaves^10,15^ and can be associated with lower soil C storage^35^. Altogether, analogous fast-slow traits might be useful in C cycling models, but the use of the fast-slow trade-off axis as a predictor might be less reliable because of the weak relationship between root dry matter content and leaf N.

We found support for our second hypothesis that a second trait axis will consist of traits related to resource acquisition (Fig. 3). The principal component analysis showed a resource acquisition gradient with specific leaf area and the two acquisitive root traits (i.e. specific root length and specific root area) at the one end, and average root diameter at the other end. However, post-hoc Spearman’s rank correlation did not confirm a significant relationship between specific leaf area and specific root length. The finding contrasts with some previous studies that found a significant positive relationship between specific root length and specific leaf area^4,9,13^, but align with a review that found no conclusive evidence for this relationship^36^. Generally, a larger specific leaf area is expected to result in more carbon fixation^39^, and an associated higher leaf N content^15^, as N is required for the production of RUBISCO, the enzyme critical for photosynthesis. It has been hypothesized that this demand could be supplied by fine roots with higher specific root length^15^. Therefore, resource acquisition in leaves is related to light capture and subsequent carbon fixation, while root acquisition is related to nitrogen acquisition.

Interestingly, although we found no significant relationship between specific root length and specific leaf area, there was a significant negative relationship between specific leaf area and average root diameter, a root trait that has been shown to be related to mycorrhizal fungi colonization^10^ and outsourcing for nutrient uptake^18^. Boreal forest tree species have a strong dependence on ectomycorrhizal fungi for nutrient uptake as nitrogen availability is low^44^. Thus, our study suggests that conservative leaf traits might be more tightly coupled to a higher reliance on mycorrhizal fungi as a nutrient uptake pathway rather than a “do it yourself” uptake strategy. For example, lower values of specific leaf area represent a conservative leaf strategy that might drive plants to have higher dependence on ectomycorrhizal fungi for nutrient uptake. Therefore, the resource acquisition trade-off axis observed in the principal component analysis might be driven by relationships between individual conservative traits and average root diameter. Our study is the first, to the best of our knowledge, to find some evidence for an above-belowground resource acquisition axis. This is likely because the study by Weigelt et al.^17^. did not include specific leaf area, but rather leaf mass area, the inverse trait of specific leaf area, as the analogous trait of root tissue density.

We found partial support for our third hypothesis that species identity would explain the largest proportion of the total trait variation, followed by between and then within-site variation. We found that species identity did indeed explain the largest percentage of trait variation across all plots. This is consistent with the findings of recent studies^28,30^, and points to the importance of phylogenetic conservatism in driving trait variation^16^. However, contrary to our expectations, between site differences explained very little to none of the total variation for all measured traits. Given that the sites differed substantially in many aspects, including their annual precipitation, temperature and soil N concentration, such little variation explained by site was surprising. Climatic and soil factors have recently been shown to explain the two-dimensional spectrum of global plant trait variation, which is comprised of aboveground traits^45^. Belowground, environmental factors related to site differences, such as soil nutrient availability, mean annual temperature and mean annual rainfall has been found to influence specific root area and specific root length^5,20,22^, while others have been inconsistent or inconclusive across studies. Here, site did contribute to some of the total trait variation for root traits related to N content and the collaboration gradient (i.e. specific root length, specific root area and average diameter). This was likely driven by the differences in soil N at the two sites. However, strong relationships between root trait values and environmental factors remain elusive even with large variation in environmental factors^25^.

Interestingly, the second largest contributor to the total trait variation was within-site variation (i.e. differences between the blocks). The source of this variation could be microclimatic variation, fine-scale environmental variation, or genetic variability within the population of each species considered. It is therefore plausible that these sources of within-site variation may have a stronger influence on trait variation than macroclimatic differences between sites. For example, a recent study by Kemppinen & Niittynen^46^ demonstrated the importance of microclimatic factors such as soil moisture and snow conditions for driving intraspecific variation in specific leaf area and leaf dry matter content in sub-arctic plants. Site heterogeneity (including fine-scale soil nutrient availability) has also been shown to influence trait variation at the individual species and community levels^47,48^. However, the majority of studies examining within-site variation on total trait variation have focused on aboveground traits or few root traits^23,24^, and there are few studies in forest ecosystems.

Our study goes one step further and compares trait variation across a large number of species both within and between sites, and includes both root and leaf traits. In doing so, we show that within-site variation plays an important role in explaining total trait variation across boreal forest tree species, more so for morphological root traits than leaf traits. This is in line with recent findings from Kumordzi et al.^26^. for boreal understorey shrubs, where experimental plots contributed to a larger percentage of the total variation of specific root length relative to multiple leaf traits measured. This may be because differences in fine-scale heterogeneity and soil microclimatic variables directly interact with roots and not leaves. These factors can influence the strength of above- and belowground trait coordination found across species^49^, and could be a factor contributing to some disagreement on such relationships in existing literature^11,17,18^. However, we found no obvious link between the relative contributions of the sources of trait variation measured in this study and whether or not analogous above- and belowground traits were correlated with each other.

Boreal forests cover approximately 17% percent of terrestrial land surface and account for approximately 20–40% of terrestrial C stocks^33,34,50^. Therefore, understanding the trait variation and relationships can improve on global and regional C cycling models. This is because missing data on either above- or belowground trait variation could be substituted by analogous traits that have significant positive or negative relationships. Further, coordinated fast-slow economic strategies and resource acquisition strategies in roots and leaves of trees could improve ecosystem models on tree effects on C cycling. For example, fast-slow strategies influence microbial community composition and organic matter decomposition rates^35^, and both higher specific leaf area and thicker fine root diameters influence root exudation fluxes^51,52^ and therefore long-term C storage^53^. However, this fine scale variation introduces some uncertainty into the macro-scale patterns that should be incorporated into macro scale models.

## Materials and methods

### Site description and sampling design

Two common garden experiments, one each in northern and central Sweden, Svartberget (64°15′N 19°47′E) and Garpenberg (60°18′N 16°17′E), which are approximately 590 km apart, were utilized for this experiment (Fig. 1). The sites were established in 1992 and 1995, respectively, to assess the growth of potential relative to current commercial boreal tree species in Sweden. Both sites consist of three replicate blocks, each consisting of plots with monocultures of tree species from the boreal forest region. At Svartberget, there are 10 tree species, while at Garpenberg there are 8 tree species (Fig. 1). Altogether, the sites comprise of more than 50% of all boreal tree species. Six of the species are common between the two sites. At both sites, within each experimental block there are plots (0.1 ha) consisting of monocultures of each tree species planted at the respective experimental site. However, a few species did not have three replicate plots at the southern site (Garpenberg) (Fig. 1). This resulted in a total of 30 plots at Svartberget (with a total area of 3 ha) and 20 plots at Garpenberg (with a total area of 2 ha). With the exception of *Betula pendula*, *Pinus sylvestris*,* Larix sukaczewii* and *Picea abies*, all other tree species are not native to Sweden, but are common in boreal forest ecosystems. The two common garden sites exhibit several environmental differences in temperature and nutrients (Table S2). The soil properties for each species at both sites are found in Table S3. These soil property data were obtained from soil samples collected during the growing season in 2021. The organic horizon was systematically sampled in a grid pattern at 10 locations in each plot (i.e. sub-samples) using a PVC tube (Ø10 cm) fitted with a serrated blade. Sub-samples were then pooled and sieved (Ø 2 mm), homogenized, and dried at 70 °C for 48 h, and then ground with a Retch MM400 ball mill. Total carbon (C) and nitrogen (N) concentrations were analysed by dry combustion using an elemental analyser (Flash EA 2000; Thermo Fisher Scientific, Bremen, Germany).

### Sample collection and processing

Leaf and root samples were collected in August 2022 from each plot at both common garden sites, and processed according to protocols outlined in Pérez-Harguindeguy et al.^54^. Briefly, one twig was collected from the sun-exposed canopy of three random trees in each plot using a 15 m long telescopic pruner. The base of the twigs were immediately wrapped with tissue sprayed with de-ionized water and pooled in a ziploc bag. The bags were filled with air, sealed, and stored in a cooler. Thereafter, samples were stored at 4 °C for a maximum of 1 ˗ 2 days after which they were further processed and scanned. Prior to scanning, six healthy green leaves of *Betula pendula* and *Pinus* species that had no sign of herbivory were selected and placed in separate aluminium trays for 30 min to rehydrate. Leaves from *Abies lasciocarpa*, *Pseudotsuga menziesii*, *Picea* and *Larix* species were processed similarly, but instead twenty four needles were removed and scanned. The larger number of needles for these species was selected because their leaves were much smaller than those of the former species. For coniferous species, needles were collected from the most recent growth year to make the values of N content comparable across all species. The leaf sub-samples were scanned using a WinRhizo 2016 with a flatbed scanner (EPSON Perfection V800/V850 1.9 V3.93 3.9.3.2) at 400 dpi resolution.

One root core (Ø10 cm) was taken from a maximum distance of 1 m from the base of four random trees in each plot^30^. The four cores from each plot were pooled into a large bag, stored in coolers for two days until transport to the laboratory, and then subsequently refrigerated at 4 °C until further processing. Roots were carefully washed over a sieve (4 mm) and a tray, and the flow-through from the tray was poured over a 2 mm sieve to recover any roots that were accidentally broken during rinsing between successive rinses. We then washed off any remaining soil particles on the roots with light water spraying. Roots from understorey vegetation were discarded. A representative fine root (< 2 mm diameter) sub-sample from each washed core was selected for scanning. While this diameter class is frequently used in trait studies^30,41^, it is likely that this diameter-based selection of fine roots resulted in a combination of both absorptive and transport roots^55,56^. The sub-samples from all four of the washed cores were subsequently pooled and two scans of these pooled roots were subsequently performed. We scanned the roots in transparent trays (15 × 20 cm) with cold tap water using the scanner above at 800 dpi resolution, with overhead lights on, and detection of very pale roots selected. We considered the tray area as a method of standardization for the quantity of roots scanned across plots, as trays were filled with roots without overlapping. The trait values were subsequently averaged to obtain an average trait value per plot. Root and leaf traits were not measured on the same individuals in each plot, as each 0.1 ha monoculture plot was considered the unit of replication for trait measurements.

We selected a suite of analogous leaf and root traits that have relevance to either the “leaf economics spectrum” or the “root economics space”. Chemical trait data were obtained by first manually grinding a sub-sample (approx. 150 mg) of the scanned dried leaf or fine root material from each of the same individuals from which we obtained the scanned samples. Samples were ground with a Retch MM400 ball mill. We analysed total C and N concentrations by dry combustion using an elemental analyzer (Flash EA 2000, Thermo Fisher Scientific, Bremen, Germany). Regarding morphological root traits for each scanned root subsample, we measured total root length and root surface area. We then recorded the fresh weight of each scanned sample and the dry weight after drying at 60 °C for two days. We subsequently used the total biomass of the sample to calculate specific root length (cm g^− 1^) and specific root area (cm^2^ mg^− 1^) and root dry matter content (dry mass per unit fresh mass; mg mg^− 1^). Similarly, mass-dependent leaf traits, i.e. specific leaf area and leaf dry matter content were calculated using the total biomass of the scanned samples.

### Data analysis

All statistical analyses were carried out using R 4.2.1 (R Core Team 2022, Vienna, Austria). For all statistical analysis, we considered individual plots as the unit of replication (50 plots in total). Data were aggregated to the plot level by either compositing sub-samples prior to measurements, or by averaging sub-plot data to the plot level after measurements were performed. Prior to performing statistical analyses, all trait data were log-transformed. The assessment of fine root trait variation and the leaf trait variation across the twelve tree species were performed using two separate Principal Component Analyses (PCA) to confirm that aboveground and belowground trait relationships are in line with those in the literature. Subsequently, to assess the coordination of analogous above- and belowground traits the datasets were pooled and a third PCA was performed. We conducted a Horn’s test of parallel analysis that is used to determine the number of dimensions required to explain the variation in a multidimensional trait dataset (as in Carmona et al.^11^). using the package “paran” and with 9999 iterations. If total dimensions required to explain the variation in the total dataset exceeds the sum of the individual datasets, then the datasets are not that strongly correlated. Therefore, we first conducted the analysis with all traits together, then a subset of root traits only and finally with leaf traits only. Our analysis showed that two components should be retained for the separate datasets and three components for the total dataset. Hence, the root and leaf traits are correlated.

We then performed post-hoc Spearman’s rank correlations to assess the statistical significance of relationships between the analogous root and leaf traits. Spearman’s rank correlations with Holm’s posthoc correction reduces the risk of Type I errors that could occur with multiple comparisons^57^. An additional PCA was performed, as above, to assess above- and belowground trait coordination at each site. To assess the relative contributions of between site variation, intraspecific variation and species identity to the total variation of each trait, we conducted a variance component analysis using a nested model and with the varcomp() function as in Liu et al.^28^. Briefly, for each measured trait we performed a general linear model with only random factors in the nested structure of site/species/block. Species were nested in site because there are several species present at each site, some of which are not common between the sites. This accounts for differences between the sites and interspecific trait variation. Block was further nested under species to account for replicates of each species (i.e. intraspecific trait variation). The error represented the remaining unexplained trait variation (i.e. residual variation).

## Electronic supplementary material

Below is the link to the electronic supplementary material.

Supplementary Material 1

## Data Availability

All trait data for the study will be openly accessible on Dryad Digital Repository upon manuscript acceptance at https://doi.org/10.5061/dryad.4tmpg4fk8.
