# Supplementary material for: Aboveground and belowground trait coordination across twelve boreal forest tree species
Source: Sci Rep. 2025 Jan 3;15:680. doi: 10.1038/s41598-024-84162-0 (PMC11698914; doi:10.1038/s41598-024-84162-0)

**Supplementary Information**

Above- and belowground trait coordination across boreal forest tree species

**Table S1.** Relative contributions of intraspecific trait variation, site, and interspecific trait variation to total trait variation for above and belowground trait pairs. Only trait pairs with significant spearman rank correlations are included. Trait pairs with red shading have positive correlations, whereas those with blue shading have negative correlations. The six traits are leaf nitrogen content (Leaf N); specific leaf area (SLA); leaf dry matter content (LDMC); leaf carbon to nitrogen ratio (Leaf C:N); average fine root diameter (AD); root nitrogen content (Root N); specific root area (SRA); root carbon content (Root C); root carbon to nitrogen ratio (Root C:N), and root dry matter content (RDMC).

| **Source of**  **variation** | **LDMC/**  **RDMC** | **LDMC/**  **Root N** | **LDMC/**  **Root C:N** | **LDMC/**  **Root C** | **LDMC/**  **SRA** | **LDMC/**  **SRA** | **Leaf C:N/**  **Root C:N** | **Leaf C:N/**  **Root N** | **Leaf N/**  **Root N** | **Leaf N/**  **Root C:N** | **Leaf N/**  **Root C** | **Leaf C:N/**  **Root C** | **SLA/**  **AD** |
| --- | --- | --- | --- | --- | --- | --- | --- | --- | --- | --- | --- | --- | --- |
| Intraspecific | 14.3%**/**  46.0% | 14.3%/  23.2% | 14.3%/  23.7% | 14.3%/  34.1% | 14.3%/  22.1% | 14.3%/  22.1% | 27.2%/  23.7% | 27.2%/  23.2% | 25.3%/  23.2% | 25.3%/  23.7% | 25.3%/  34.1% | 27.2%/  34.1% | 1.6%/  37.2% |
| Site | 0%/  0% | 0%/  5.9% | 0%/  6.9% | 0%/  0% | 0%/  3.1% | 0%/  3.1% | 0%/  6.9% | 0%/  5.9% | 0%/  5.9% | 0%/  6.9% | 0%/  0% | 0%/  0% | 0%/  2.6% |
| Interspecific | 85.5%/  53.8% | 85.5%/  64.5% | 85.5%/  63.1% | 85.5%/  65.7% | 85.5%/  68.9% | 85.5%/  68.8% | 72.6%/  63.1% | 72.6%/  64.5% | 74.5%/  64.5% | 74.5%/  63.1% | 74.5%/  65.7% | 72.6%/  65.7% | 98.2%/  52.7% |

**Table S2**. Environmental properties of the two study sites. Average growing season temperature during 2018 – 2022 was calculated from data obtained from the Swedish Meteorological and Hydrological Institute. Soil properties are across-plot averages.

| **Environmental variable** | **Svartberget** | **Garpenberg** |
| --- | --- | --- |
| Average temperature | 13.3°C | 15.9°C |
| Soil C | 29.72% | 45.1% |
| Soil N | 0.90% | 1.78% |
| Soil C:N | 33.8% | 25.5% |
| Soil pH | 4.24 | 3.96 |

**Table S3**. Soil properties at the two common garden sites for each species.

| **Site** | **Species** | **% C** | **% N** | **C:N ratio** | **pH** |
| --- | --- | --- | --- | --- | --- |
| Garpenberg | *Picea abies* | 46.69 | 1.69 | 27.7 | 3.89 |
|  | *Picea glauca* | 44.84 | 1.71 | 26.46 | 3.91 |
|  | *Pinus sylvestris* | 45.73 | 1.75 | 26.16 | 3.88 |
|  | *Larix sukaczewii* | 45.84 | 1.81 | 25.30 | 4.10 |
|  | *Larix sp.* | 45.66 | 1.85 | 24.70 | 3.87 |
|  | *Pseudotsuga menziesii* | 45.32 | 1.86 | 24.53 | 4.10 |
|  | *Betula pendula* | 44.20 | 1.91 | 23.20 | 3.97 |
|  | *Pinus contorta* | 42.14 | 1.65 | 25.60 | 3.96 |
| Svartberget | *Picea abies* | 24.19 | 0.67 | 35.40 | 4.21 |
|  | *Picea glauca* | 31.20 | 1.00 | 31.40 | 4.37 |
|  | *Pinus sylvestris* | 30.25 | 0.97 | 31.33 | 4.33 |
|  | *Larix sukaczewii* | 27.80 | 1.00 | 28.5 | 4.32 |
|  | *Betula pendula* | 27.61 | 0.96 | 28.93 | 4.62 |
|  | *Pinus contorta* | 30.02 | 0.87 | 34.87 | 4.23 |
|  | *Picea mariana* | 36.03 | 0.97 | 38.7 | 3.95 |
|  | *Pinus banksianna* | 27.41 | 0.89 | 31.73 | 4.39 |
|  | *Abies sibirica* | 27.03 | 0.77 | 35.70 | 4.08 |
|  | *Abies lasciocarpa* | 35.64 | 0.87 | 41.23 | 3.86 |

**Table S4.** Average trait values across all tree species, as well as their standard deviations (sd) and coefficients of variation at both sites (CV Both), at Svartberget (CV Svartberget) and at Garpenberg (CV Garpenberg). The five leaf traits are leaf carbon content (Leaf C); leaf nitrogen content (Leaf N); specific leaf area (SLA); leaf dry matter content (LDMC); leaf carbon to nitrogen ratio (Leaf C:N). The seven fine root traits are average fine root diameter; root nitrogen content (Root N); specific root area (SRA); specific root length (SRL); root carbon content (Root C); root carbon to nitrogen ratio (Root C:N), and root dry matter content (RDMC).

| **Trait** | **Unit** | **Mean Both** | **Mean Svartberget** | **Mean Garpenberg** | **CV Both** | **CV Svartberget** | **CV Garpenberg** |
| --- | --- | --- | --- | --- | --- | --- | --- |
| Leaf C:N | - | 45.09  (11.83) | 46.45  (11.50) | 43.07  (12.34) | 26.25 | 24.76 | 28.65 |
| Root C:N | - | 56.67  (12.24) | 59.08  (14.13) | 53.06  (7.67) | 21.61 | 23.92 | 14.46 |
| Leaf C | % | 51.10  (1.57) | 50.85  (1.87) | 51.48  (0.87) | 3.07 | 3.67 | 1.70 |
| Root C | % | 52.64  (1.07) | 52.78  (1.17) | 52.43  (0.91) | 2.04 | 2.21 | 14.46 |
| LDMC | g^-1^g^-1^ | 0.41  (0.05) | 0.42  (0.05) | 0.41  (0.04) | 11.59 | 11.67 | 11.57 |
| RDMC | g^-1^g^-1^ | 0.29  (0.04) | 0.29  (0.04) | 0.30  (0.04) | 12.66 | 13.08 | 11.89 |
| Leaf N | % | 1.23  (0.41) | 1.19  (0.40) | 1.30  (0.42) | 33.07 | 33.91 | 31.97 |
| Root N | % | 0.96  (0.17) | 0.93  (0.18) | 1.01  (0.14) | 17.26 | 18.91 | 14.17 |
| Root diameter | mm | 0.78  (0.09) | 0.76  (0.08) | 0.80  (0.08) | 11.04 | 11.14 | 10.25 |
| SLA | cm^2^ g^-1^ | 52.23  (42.88) | 53.26  (47.98) | 50.69  (34.97) | 82.09 | 90.09 | 69.00 |
| SRA | cm^2^ g^-1^ | 155.30  (40.89) | 165.02  (39.55) | 140.72  (39.39) | 26.32 | 23.97 | 27.99 |
| SRL | cm g^-1^ | 659.7 | 714.6 | 577.5 | 36.4 | 33.4 | 38.8 |
|  |  | (240.3) | (238.5) | (224.0) |  |  |  |

**Table S5**. Average trait values by species across both sites. The tree species are *Abies lasciocarpa* (AL); *Abies sibirica* (AS); *Betula pendula* (BI); *Pinus contorta* (CO); *Pseudotsuga menziesii* (DO); *Larix sp*. (LH); *Larix sukaczewii* (LS); *Pinus banksianna* (PB); *Picea mariana* (PM); *Pinus sylvestris* (PN); *Picea abies* (SP). and *Picea glauca* (WS). The five leaf traits are leaf carbon content (C); leaf nitrogen content (N); specific leaf area (SLA); leaf dry matter content (LDMC); leaf carbon to nitrogen ratio (C:N). The seven fine root traits are average fine root diameter (AD); root nitrogen content (N); specific root area (SRA); specific root length (SRL); root carbon content (C); root carbon to nitrogen ratio (C:N). and root dry matter content (RDMC).

| **Species** | **Leaf C** | **Leaf N** | **Leaf C:N** | **SLA** | **LDMC** | **AD** | **SRL** | **SRA** | **RDMC** | **Root C** | **Root N** | **Root C:N** |
| --- | --- | --- | --- | --- | --- | --- | --- | --- | --- | --- | --- | --- |
| AL | 51.37 | 0.96 | 53.79 | 33.57 | 0.18 | 0.94 | 616.22 | 139.48 | 0.33 | 54.24 | 0.63 | 87.36 |
| AS | 51.17 | 0.95 | 54.09 | 40.03 | 0.14 | 0.98 | 512.48 | 125.17 | 0.31 | 53.29 | 0.75 | 71.79 |
| BI | 50.30 | 2.04 | 24.73 | 167.03 | 0.78 | 0.83 | 1109.76 | 219.84 | 0.31 | 53.60 | 1.12 | 48.16 |
| CO | 51.30 | 1.07 | 48.45 | 34.98 | 0.38 | 1.20 | 781.82 | 190.43 | 0.24 | 51.59 | 1.10 | 47.39 |
| DO | 52.06 | 1.38 | 38.82 | 45.06 | 0.17 | 0.68 | 384.10 | 102.81 | 0.33 | 52..88 | 0.86 | 61.63 |
| LH | 50.90 | 1.60 | 35.38 | 88.99 | 0.10 | 1.03 | 658.67 | 158.40 | 0.27 | 51.92 | 1.02 | 51.15 |
| LS | 50.63 | 1.75 | 29.41 | 63.49 | 0.25 | 1.08 | 621.31 | 157.30 | 0.26 | 51.96 | 1.15 | 45.36 |
| PB | 50.98 | 1.10 | 46.42 | 26.03 | 0.29 | 1.58 | 769.36 | 184.34 | 0.28 | 50.93 | 0.94 | 53.81 |
| PM | 52.16 | 0.98 | 53.04 | 33.00 | 0.11 | 0.81 | 994.78 | 201.12 | 0.30 | 53.48 | 0.85 | 63.29 |
| PN | 51.49 | 1.23 | 41.93 | 41.40 | 0.20 | 1.00 | 576.77 | 139.87 | 0.29 | 51.87 | 0.96 | 54.93 |
| SP | 50.50 | 0.92 | 55.80 | 30.82 | 0.13 | 0.72 | 456.22 | 115.49 | 0.34 | 53.48 | 0.86 | 62.78 |
| WS | 51.07 | 0.97 | 54.10 | 28.06 | 0.15 | 1.14 | 485.81 | 131.07 | 0.29 | 52.70 | 1.05 | 50.78 |

**Table S6.** Spearman rank correlations of a subset of paired traits across six species common between the two study sites. as well as of each species. The traits are root dry matter content (RDMC); leaf dry matter content (LDMC); leaf nitrogen content (Leaf N); root nitrogen content (Root N); specific leaf area (SLA). and specific root length (SRL). Red shading showing strong positive correlations. whereas blue shading shows negative correlations. Unshaded cells are weak correlations.

| **Trait pair** | **Across six species** | ***P. sylvestris*** | ***P. abies*** | ***P. glauca*** | ***P. contorta*** | ***B. pendula*** | ***L. sibirica*** |
| --- | --- | --- | --- | --- | --- | --- | --- |
| RDMC-LDMC | 0.30 | 0.09 | 0.49 | 0.60 | – 0.77 | 0.70 | 0.20 |
| Leaf N - Root N | 0.43 | 0.49 | 0.03 | 0.94 | – 0.14 | – 0.10 | 0.21 |
| RDMC- Leaf N | – 0.17 | – 0.09 | – 0.09 | – 0.77 | 0.60 | – 0.36 | – 0.74 |
| LDMC - Root N | – 0.15 | 0.49 | 0.29 | – 0.26 | 0.09 | 0.60 | – 0.40 |
| SRL - SLA | 0.62 | 0.60 | – 0.49 | – 0.37 | 0.14 | – 0.20 | 0.80 |
| AD - SLA | – 0.53 | – 0.31 | 0.77 | – 0.20 | – 0.03 | 0.30 | 0.40 |

**Figure S1**. PCA by site. The six leaf traits are leaf carbon content (Leaf C); leaf nitrogen content (Leaf N); specific leaf area (SLA); leaf dry matter content (LDMC); leaf carbon to nitrogen ratio (Leaf C:N). The eight fine root traits are average fine root diameter; root nitrogen content (Root N); specific root area (SRA); specific root length (SRL); root carbon content (Root C); root carbon to nitrogen ratio (Root C:N). and root dry matter content (RDMC).


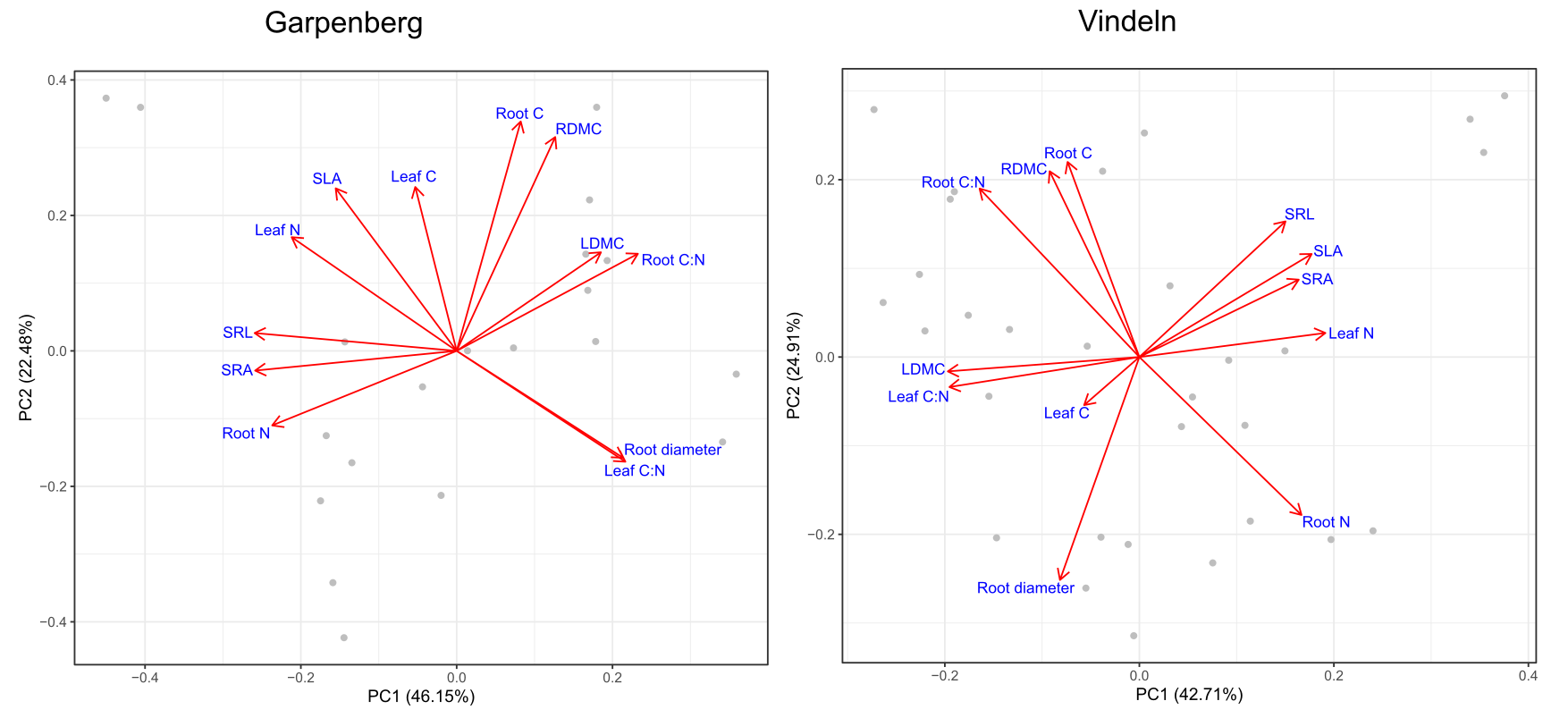

Supplement: Supplementary file 1 — Supplementary Material 1 [file 41598_2024_84162_MOESM1_ESM.docx]
